# Sub-MIC Antibiotics Modulate Productions of Outer Membrane Vesicles in Tigecycline-Resistant *Escherichia coli*

**DOI:** 10.3390/antibiotics13030276

**Published:** 2024-03-19

**Authors:** Qianru Li, Jun Li, Tao He, Xing Ji, Ruicheng Wei, Meiling Yu, Ran Wang

**Affiliations:** 1 School of Animal Science and Technology, Guangxi University, Nanning 530004, China; 2118302019@st.gxu.edu.cn; 2 Key Laboratory of Food Quality and Safety of Jiangsu Province-State Key Laboratory Breeding Base, Key Laboratory of Agro-Product Safety Risk Evaluation (Nanjing) of Ministry of Agriculture and Rural Affairs, Institute of Food Safety and Nutrition, Jiangsu Academy of Agricultural Sciences, Nanjing 210014, China; 20180040@jaas.ac.cn (J.L.); 20150021@jaas.ac.cn (T.H.); 20210099@jaas.ac.cn (X.J.); rcwei79@jaas.ac.cn (R.W.)

**Keywords:** outer membrane vesicle, tigecycline, *tet*(X4), transcriptome, SOS response

## Abstract

Antimicrobial resistance (AMR) has been recognized as one of the most important crises affecting global human health in the 21st century. Tigecycline is one of the last resort antibiotics for treating severe infections caused by multi-drug resistant *Enterobacteriaceae*. However, the mobile resistance gene *tet*(X4), which could mediate high-level tigecycline resistance, was discovered in 2019. The outer membrane vesicle (OMV) has been recognized as a new route for horizontal gene transfer; antimicrobial resistant bacteria also have the ability to secret OMVs, while little is known about the impact of antibiotics on the secretion and characteristics of OMVs from tigecycline resistant bacteria till now. This study aimed to investigate the effects of antibiotics on the production and traits of a tigecycline resistant *Escherichia coli* strain of 47EC. The results showed that sub-inhibitory (1/2 MIC or 1/4 MIC) concentrations of gentamicin, meropenem, ceftazidime, chloramphenicol, tigecycline, ciprofloxacin, polymycin, rifaximin and mitomycin C could significantly increase the secretion of OMVs (0.713 ± 0.05~6.333 ± 0.15 mg/mL) from *E. coli* 47EC compared to the respective untreated control (0.709 ± 0.03 mg/mL). In addition, the particle sizes of OMVs were generally larger, and the zeta potential were lower in the antibiotics-treated groups than those of the antibiotic-free group. The copy numbers of the tigecycline resistance gene of *tet*(X4) in the OMVs of most antimicrobial-treated groups were higher than that of the control group. Moreover, transcriptome analysis on ciprofloxacin-treated *E. coli* 47EC indicated that the SOS response and prophage activation might participate in the ciprofloxacin-induced OMV formation. In conclusion, the clinical application of antibiotics in treating bacterial infections, especially multi-drug resistant bacteria, might lead to the increased secretion of bacterial OMVs and the enrichment of antimicrobial-resistant genes in the OMVs.

## 1. Introduction

Antimicrobial resistance is an increasingly severe threat to global public health [1]. If no action is taken, the human deaths caused by antibiotic-resistant bacteria are estimated to be 10 million per year by 2050, which is 1.8 million more than cancer death [2]. The multi-drug-resistant bacteria, particularly those resistant to last-resort drugs (carbapenems, colistin and tigecycline) cause the most serious concern [3].

Tigecycline, a third-generation semi-synthetic tetracycline, has been developed for the treatment of polymicrobial infections caused by Gram-positive and Gram-negative pathogens and is classified as one of the critically important antibacterial (CIA) drugs by the WHO (World Health Organization) [4]. Resistance to tigecycline is mediated by different mechanisms including ribosomal protein binding site mutation, cell membrane variation, efflux pump transport system activation and modification enzyme degradation [5]. In 2019, a novel plasmid-encoded transmissible *tet*(X4) gene, which could confer high-level tigecycline resistance, was discovered in an *Enterobacteriaceae* isolate from animals in China [6,7]. The *tet*(X4) gene encodes a FAD-development monooxygenase that could regioselectively hydroxylate tetracycline substrates. Most of the *tet*(X4) genes are located on different types of plasmids such as IncQ1, IncX1, IncFIB, IncHI1 and hybrid plasmids [5].

The outer membrane vesicle is a newly found mechanism of horizontal gene transfer apart from transformation, conjugation and transduction [8,9]. OMVs are 20- to 400-nm spherical bilayer liposomes that are naturally secreted by bacteria during growth [10,11]. OMVs could enclose the cargos of lipopolysaccharide (LPS), phospholipids, genetic elements (DNA, RNA, and plasmids), and periplasmic as well as cytoplasmic protein components [12]. The outer membrane vesicles have diverse functions, such as protein transport, horizontal gene transfer, nutrient acquisition, cell-intercellular communication, antibacterial activity, toxin delivery and host-immune response modulation [13,14]. The biogenesis of OMVs was influenced by many factors, such as pH, temprature, hypoxia, nutrition-deficient, etc., especially antibiotic stress. For instance, Mi Hyun Kim et al. found that *Enterococcus faecium* ATCC700221 cultured with 1/2 MIC of vancomycin or linezolid produced 3.0 and 1.5 times more membrane vesicles than bacteria cultured without antibiotics, respectively [15]. Simon Devos et al. reported that treatment with the β-lactam antibiotic imipenem led to a dramatic increase in the secretion of OMVs in the nosocomial pathogen *Stenotrophomonas maltophilia* [16]. 

Antimicrobial resistant bacteria have been reported to have the ability to secret OMVs [8], but the production under antibiotic stress conditions and the characteristics thereof have yet to be determined. The aim of this study was to investigate the production and traits of OMVs in a tigecycline resistant *E. coli* strain of 47EC under exposure of sub-inhibitory concentrations of different antibiotics.

## 2. Results

### 2.1. AMR Phenotypes of E. coli 47EC

The AMR phenotype of *E. coli* 47EC was determined using the broth microdilution method according to the CLSI M100 standard, 28th Edition [17]. The antimicrobial susceptibility testing results showed that the MICs of the control strain ATCC25922 were all within the quality control ranges (Table 1), indicating the validity of the MIC tests. *E. coli* 47EC was resistant to tigecycline, chloramphenicol and rifaximin, mediate resistant to gentamicin, and sensitive to ceftazidime, ciprofloxacin, polymyxin B and meropenem. 

### 2.2. Effects of Antibiotics on the Production of OMVs from E. coli 47EC

**Figure 1:**
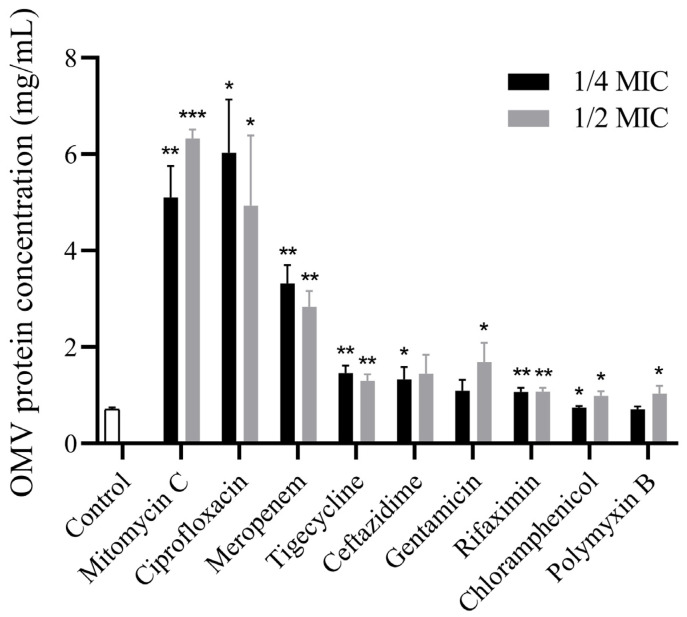
The protein concentrations of OMVs isolated from *E. coli* 47EC treated with different antibiotics. Each test was performed with three independent repeats, and the error bars show the standard deviations (SDs). The significant difference of the protein concentrations between the antibiotic-treated groups and the antibiotic-free group was tested using the unparied *t* test; * *p* < 0.05; ** *p* < 0.01; *** *p* < 0.001.

To investigate the effects of different antibiotics on the production and characteristics of OMVs, the bacterial cultures of *E. coli* 47EC were treated with gentamicin, meropenem, ceftazidime, chloramphenicol, tigecycline, ciprofloxacin, polymycin, rifaximin and mitomycin C at sub-inhibitory concentrations (1/2 MIC or 1/4 MIC). The OMVs were extracted from *E. coli* 47EC treated with or without antibiotics by using the ultracentrifugation method. The BCA results showed that the protein concentration of OMVs without antibiotic treatment was 0.709 ± 0.03 mg/mL (Figure 1). After being treated with different antibiotics, the productions of OMVs from *E. coli* 47EC were significantly increased (0.713 ± 0.05~6.333 ± 0.15 mg/mL). The positive control mitomycin C exhibited the strongest stimulation effect on the production of OMVs. Among the different antibiotics, the concentrations of OMVs in the ciprofloxacin group (1/2 MIC: 4.938 ± 1.19 mg/mL and 1/4 MIC: 6.03 ± 0.90 mg/mL) were relatively higher than in other antibiotic groups. 

### 2.3. Effects of Antibiotics on the Characterizations of OMVs from E. coli 47EC

**Figure 2:**
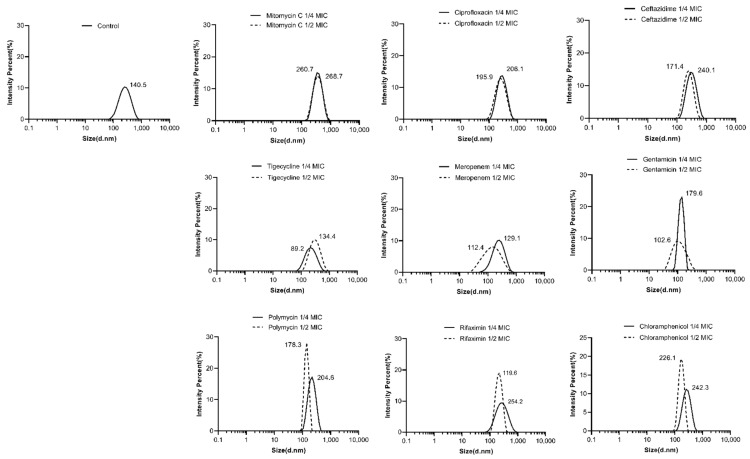
The particle size distribution of the OMVs isolated from *E. coli* 47EC and treated with different antimicrobials.

The DLS-intensity weighted particle size distribution of the OMV was determined using a Zeta-sizer nano-ZS (Malvern Instruments, Worcestershire, UK). The particle size of the OMV without antibiotic treatment was 140.5 nm (Figure 2). After being treated with antibiotics such as mitomycin C, ciprolfoxacin, ceftazidime, polymycin, chloramphenicol, gentamicin (at 1/4 MIC) and rifaximin (at 1/4 MIC), the particle sizes of the OMVs were increased (178.3~268.7 nm), whereas other antimicrobialssuch as tigecycline, meropenem, gentamicin (at 1/2 MIC) and rifaximin (at 1/2 MIC) induced smaller OMVs. Most of the antimicrobials induced larger-size OMVs at concentrations of 1/4 MICs than at 1/2 MICs except tigecycline, whose OMVs sizes were 137.4 nm at 1/2 MIC and 89.2 nm at 1/4 MIC.

**Figure 3:**
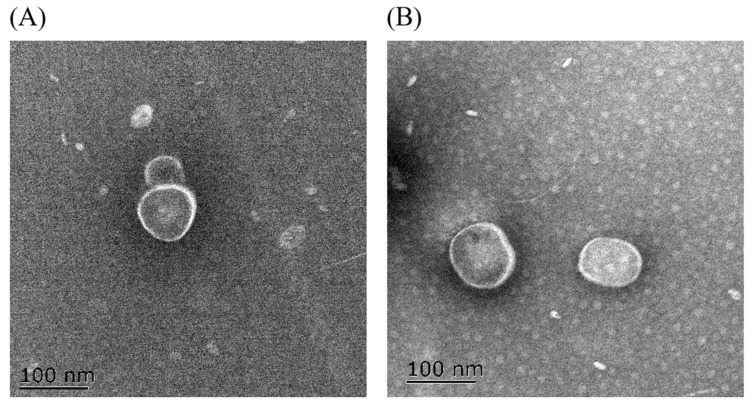
The morphology of the OMVs observed by TEM. (**A**) Without antibiotic treatment group and (**B**) Ciprofloxacin treatment group. Scale bars: 100 nm.

The differences in the size of the OMVs under antibiotics treatment were also demonstrated by the OMV morphology observed by TEM. TEM showed that the OMVs were spherical (Figure 3). The size of the OMV from the ciprofloxacin-treated group was slightly larger than that of the control group.

**Figure 4:**
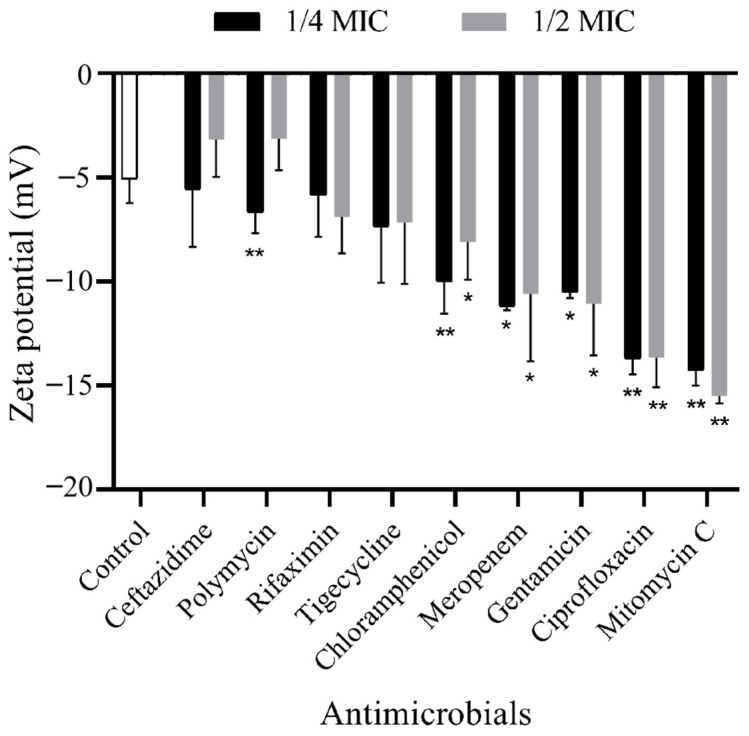
The zeta potential of the OMVs isolated from *E. coli* 47EC and treated with different antibiotics. Each test was performed with three independent repeats, and the error bars show the standard deviations (SDs). The significant difference of the zeta potentials between the antibiotic-treated groups and the antibiotic-free group was tested using the unparied *t* test; * *p* < 0.05; ** *p* < 0.01.

The zeta potential of the OMV in the control group was −5.057 ± 0.956 (Figure 4). When treated with rifaximin, tigecycline, chloramphenicol, meropenem, gentamicin, ciprofloxacin, mitomycin C, ceftazidime (1/4 MIC) and polymycin (1/4 MIC), the zeta potentials of the OMVs were decreased to −5.6 ± 2.229~−14.725 ± 1.366. Only in the groups of ceftazidime (1/2 MIC, −3.167 ± 1.456) and polymycin (1/2 MIC, −3.126 ± 1.243) were the zeta potentials of the OMVs higher than that of the antibiotic-free group. 

### 2.4. Abundance of tet(X4) in OMVs

**Figure 5:**
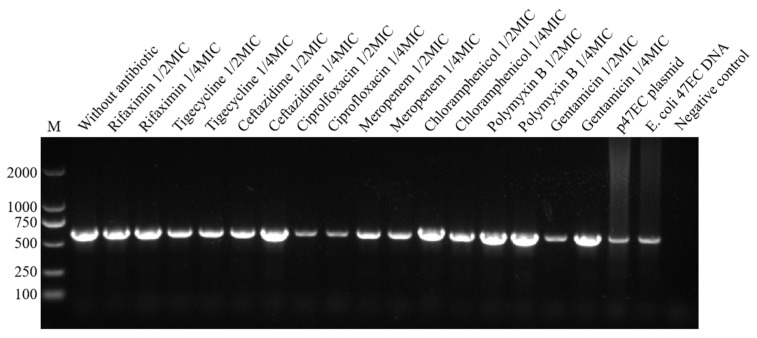
PCR detection of the tigecycline-resistant gene *tet*(X4) in the OMVs from *E. coli* 47EC in the different antibiotic treatment groups and the control group. Plasmid 47EC and the genome DNA extracted from *E. coli* 47EC bacteria were used as positive controls. Sterile water was used as the negative control.

**Figure 6:**
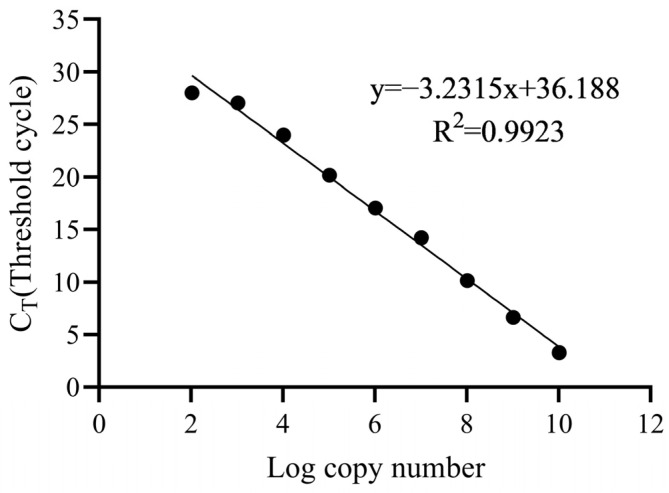
Construction of the standard curve of the quantitative PCR to determine the copy numbers of *tet*(X4) in the OMVs.

The existence of the tigecycline-resistant gene *tet*(X4) in the OMVs was examined by PCR, and the copy numbers of *tet*(X4) were quantified by qPCR. The PCR result confirmed that the OMVs originating from either the control group or the antibiotics-treated groups all contained the resistant gene of *tet*(X4) (Figure 5). The standard curve for *tet*(X4) was linear (R^2^ > 0.99) at the tested range of 1 × 10^2^ to 1 × 10^10^ copies/µL (Figure 6). The absolute qPCR reuslts showed that the copies of *tet*(X4) in the OMV of the control group was 2.937 × 10^4^ copies/µL, and the copy numbers in the OMVs from different antimicrobial-treated groups varied from 1.755 × 10^3^ to 6.875 × 10^5^ copies/µL (Table 2). For most of the antimicrobials, the copy numbers of *tet*(X4) in the OMVs were generally higher than those of the control group, expect for rifaximin and tigecycline (1/2 MIC). Also, it seemed that the copy numbers of *tet*(X4) in the OMVs were not positively correlated with the production of the OMVs induced by different antibiotics, which might be due to the different action mechanism of the antimicrobials, thereby resulting in the variation of cargos in the OMVs.

### 2.5. Transcriptome Changes in E. coli 47EC Induced by Ciprofloxacin

**Figure 7:**
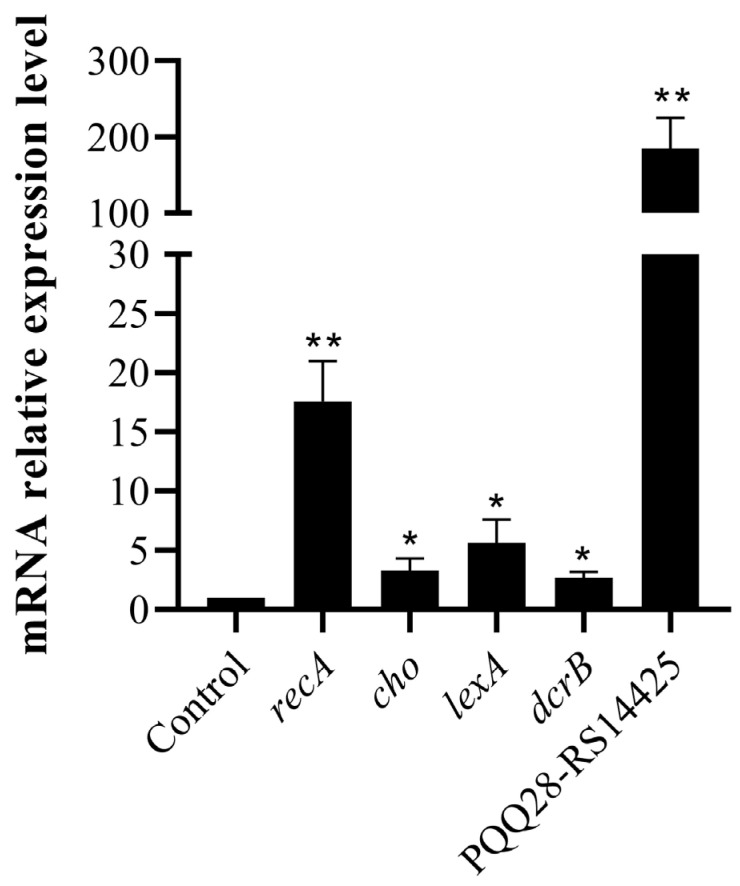
Relative expressions of *recA*, *cho*, *lexA*, *dcrB* and PQQ28-RS14425 genes in *E. coli* 47EC under treatment with ciprofloxacin. Bacteria without antibiotic treatment were taken as the control with the basal level expression indicated as 1. Data are representative of three independent experiments. Bars represent the mean ± SD. * *p* < 0.05; ** *p* < 0.01.

Because ciprofloxacin showed the strongest stimulation effects on the production of OMVs among the tested antimicrobials, we applied a transcriptome analysis to investigate the underlying mechanism. *E. coli* 47EC was treated with ciprofloxacin at a concentration of 1/2 MIC for 6 h; several changes were revealed in the transcriptomes of *E. coli* 47EC. Analysis of the RNA-seq data identified 386 genes with statistically significantly (i.e., adjusted *p* value, <0.05) altered transcription levels, with 195 genes and 191 genes showing increases and decreases of at least twofold (i.e., log_2_ > 1) in the mRNA amounts in cells growth in the presence of ciprofloxacin as compared to those grown in the absence of antibiotic, respectively. The 386 differentially expressed genes were classified into different functional categories according to the GO and the KEGG database. In the presence of ciprofloxacin, the genes coding for the proteins involved in SOS response (GO: 0009432), the cellular response to extracellular stimulus (GO: 0031668) and the DNA repair (GO: 0006281) were significantly induced, whereas the genes coding for proteins involved in the regulation of cell division (GO: 0051302), the regulation of gene expression (GO: 0010468), the regulation of cellular macromolecule biosynthetic process (GO: 2000112) and the regulation of DNA-templated transcription (GO: 0006355) were significantly repressed. At the transcription level, the SOS response, the DNA-repair-related genes (*rtcB*, *dinD*, *recA*, *recX*, *recN*, *ruvA*, *yebG*, *cho*, *ydjM*, *umuC*, *umuD*, *dinI*, *sulA*, *dinG*, *uvrB*, *yafP*, *dinB*, *polB*, *ssb1*, *uvrA*, *lexA* and *uvrD*) and the prophage-related genes (RS01710, RS14425 and RS14905) were significantly up-regulated in the ciprofloxacin-treated *E. coli* 47EC than in the untreated bacteria at different levels (Table 3). qRT-PCR was performed on five SOS response and prophage-related genes to validate the transcriptome data. The qRT-PCR results showed that the expressions of *recA*, *cho*, *lexA*, *dcrB* and PQQ28-RS14425 were increased by 17.64, 3.31, 5.68, 2.72 and 185.29-fold, respectively (Figure 7). The expression patterns of the five genes determined by qRT-PCR were found to be consistent with the transcriptome data.

It is known that quinolones, such as ciprofloxacin, kill sensitive bacteria by binding to its DNA gyrase and topoisomerase IV, leading to the formation of quinolone-gyrase-DNA complexes and resulting in bacterial DNA double-strand breaks. Several studies reported that to repair the DNA damage, bacteria would trigger the SOS response and DNA repair pathways, and this bacterial reaction would further activate prophage and promote the production of OMVs. Considering the elevated transcription of the SOS response and prophage-related genes, these pathways might participate in the formation of OMVs in the *E. coli* 47EC induced by ciprolfoxacin.

## 3. Discussion

OMVs have been recognized as a fourth pathway for horizontal gene transfer and play a key role in the dissemination of antimicrobial resistance genes [8]. However, the impact of antibiotics on the OMVs derived from antimicrobial-resistant bacteria has not been fully illustrated. To our knowledge, this is the first study investigating the modulations of different classes of antibiotics on the production and characteristics of OMVs from a tigecycline-resistant *E. coli* strain of 47EC.

This study found that treatment with different antibiotics stimulate the production of OMVs, as indicated by the protein concentration of OMVs determined by BCA. Moreover, ciprofloxacin exhibited a stronger stimulation effect than other antibiotics. The results were consistent with previous publications, showing that sub-MIC ampicillin or ciprofloxacin increased OMV secretion at 1.4–5.6-folds in *Geobacter sulfurreducens* [18]; ampicillin induction increased OMV production at 22.4-fold in a methicillin-resistant *S. aureus* [19]; imipenem and ciprofloxacin lead to dramatic increases in the OMVs secretion in *Stenotrophomonas maltophilia* [16,20]; polymyxin B, meropenem and gentamicin increased OMV production in *Pseudomonas aeruginosa* [21,22,23]; ciprofloxacin, flucloxacillin, and ceftaroline increased the OMV production in *Staphylococcus aureus* through different routes [24]. Andreas Bauwens et al. [25] reported that several antibiotics could promote OMV formation in *E. coli* strains of LB226692 (O104:H4) and 5791/99 (O157:H7) at different levels, including ciprofloxacin (250-fold and 183-fold), mitomycin C (568-fold and 470-fold), fosfomycin (77-fold and 24-fold), meropenem (27-fold and 14-fold) and polymyxin B (ninefold and sevenfold). However, gentamicin, rifaximin, tigecycline, chloramphenicol and azithromycin did not influence the OMV production [25]. Antibiotics are thought to be prominent stressors and can be considered as the most potent inducers of defense mechanisms in pathogens. The overproduction of bacterial membrane vesicles can be taken as a form of bacterial defense against stress.

In this study, the antibiotic-treated OMVs generally exhibited larger particle sizes and more negative zeta potentials as compared to the antibiotic-free group, as determined by DLS. The differences in the particle sizes of the ciprofloxacin-treated group and antibiotic-free group were also demonstrated by the OMV morphology observed by TEM. The results were consistent with other reports. Fang et al. [18] detected that the mean size of the ciprolfoxacin-treated OMVs (63.69 ± 0.2 nm) and the ampicillin-treated OMVs (62.37 ± 0.1 nm) were larger than the antibiotic-free group (60.28 ± 0.2 nm) in *Geobacter sulfurreducens.* By using TEM, they also observed some double-layer OMVs with larger sizes in addition to the classic OMVs in the ciprofloxacin-induced group. Shweta Fulsundar et al. [26] also found that gentamicin (0.1 and 0.3 μg/mL) and chloramphenicol (1 μg/mL) led to a significant increase in the diameters of the OMVs from *Acinetobacter baylyi*. In addition, OMVs from gentamicin (0.1 μg/mL)-treated cells had significantly more negative zeta potentials (−27.6 ± 0.7 mV, *p* < 0.001) than the respective control (−19.4 ± 0.4 mV). However, chloramphenicol treatment resulted in OMVs with significantly less negative surface charges (−11.2 ± 1.1 mV, *p* < 0.001) than those of the OMVs from untreated cells. Another study performed on carbapenem-resistant hypervirulent *Klebsiella pneumoniae* showed that meropenem-induced OMVs were larger (78.8 to 396 nm, median size of 161.77 nm) than the OMV without antibiotic treatment (68.1 to 396 nm, median size 147.30 nm) [27]. The differences in the production and the characteristics (size distribution and zeta potential) of the OMVs between our study and previous publications might be due to the distinctions in the bacterial species and strains, the antibiotic treatment concentration and duration, the OMV isolation and experimental methods, etc.

Reports showed that antibiotic treatment might influence the abundance of antimicrobial-resistant gens carried by the OMVs. By performing PCR, we detected the tigecycline resistant gene *tet*(X4) in all OMV groups, including the antibiotic-free group. Furthermore, qPCR results showed that most antibiotics could increase the abundance (copy numbers) of *tet*(X4) within the OMVs of *E. coli* 47EC. Similarly, Li et al. reported that the copy numbers of extended-spectrum β-lactamase (ESBL) gene *bla*_CTX-M-55_ in the OMVs from an avian pathogenic *Escherichia coli* strain of SCAO22 was 30,615 copies/µL in the enrofloxacin treatment group and 1196 copies/µL in the amoxicillin group, which were higher than the 208 copies/µL in the control group [28]. In addition to the antimicrobial-resistant genes, bacterial virulence factors such as Shiga toxin (Stx) 2a could also be enriched in the ciprolfoxacin-induced OMVs from Enterohemorrhagic *Escherichia coli* (EHEC) strains [25]. These facts indicate that the clinical usage of antibiotics for treating bacterial infections might stimulate the production of OMVs and increase the abundance of antimicrobial-resistant genes or virulence factors within the OMVs, thereby increasing the risk of them being vectors for disseminating AMR and virulence. More studies should be conducted in the future to evaluate the frequency of antibiotic-induced OMVs’ mediated horizontal gene transfer and the mechanisms of the cargo selection and packaging in the OMVs.

It has been reported that antibiotics could stimulate the production of OMVs through three pathways: envelope stress response, SOS response and inhibition of cell wall biosynthesis [29,30]. The SOS response appears to be the main mechanism for the biogenesis of OMVs [20]. Previous studies showed that ciprofloxacin could trigger bacterial SOS response to breaking the DNA double-strand, activating the prophage to encode endolysin and thus causing bacterial explosive cell lysis to produce OMVs [20,31]. Indeed, by applying a transcriptome analysis, we verified that the SOS response, DNA repair and several prophage-related genes were significantly up-regulated in the ciprofloxacin-treated *E. coli* 47EC, indicating that these pathways might participate in the ciprolfoxacin-induced OMV formation. Similarly, earlier studies reported that quinolones provoke the bacterial SOS response to DNA damage and/or to the inhibition of DNA replication and induce the *stx*-harbouring prophage activation in *E. coli* O157:H7 [32] and an enterohemorrhagic *E. coli* O104:H4 strain [33]. Recently, Fang et al., reported that ciprolfoxacin-induced OMVs contain more redox active cytochromes, facilitating iron oxides reduction [18]. Moreover, by applying electron microscopy and proteomic analysis, they also found that the SOS response triggered by ciprofloxacin induced prophage and led to the formation of outer-inner membrane vesicles (OIMVs) in the *Geobacter sulfurreducens* strain PCA. Similarly, Federica Andreoni et al. showed that ciprofloxacin induces the SOS response, triggering vesicle formation in the lysogenic strains of *Staphylococcus aureus* but not in their phage-devoid counterparts [24]. Also, the DNA amount associated with the ciprofloxacin-induced OMVs was significantly greater than that of the β-lactam antibiotic-induced OMVs. 

## 4. Conclusions

For the first time, the effects of sub-MIC concentrations of different antibiotics on the production and characteristics of OMVs were investigated in a tigecycline-resistant and *tet*(X4)-positive strain of *E. coli* 47EC. Generally, treatment with antibiotics could induce *E. coli* 47EC to secrete more OMVs with larger particle sizes and lower zeta potentials and carry more resistant genes of *tet*(X4), with the exception of a few antibiotics. The underlying mechanism of ciprofloxacin’s strong induction on the production of OMV was examined by transcriptome analysis, showing that the SOS response and prophage activation pathway might be contributing factors. Considering that the OMV plays an important role in the horizontal gene transfer, further studies should be conducted to determine whether the clinical usage of antibiotics for treating bacterial infections, especially against multi-drug resistant bacteria, would increase the risk of OMVs as vectors for disseminating AMR genes. Inhibitors for OMV production should also be developed in the future to inhibit the transmission of antimicrobial resistance.

## 5. Materials and Methods

### 5.1. Bacterial Strain

A tigecycline-resistant clinical strain of *E. coli* 47EC was isolated from a pig farm in Shandong, China in 2018. *E. coli* 47EC was used to isolate the OMVs in this study. This strain carries a plasmid of p47EC (with a size of 170,312 bp), which belongs to IncFIB(K) and harbors the tigecycline resistance gene *tet*(X4). The genome sequence of plasmid p47EC was deposited in GenBank (accession number: MK134376).

### 5.2. Antimicrobial Susceptibility Testing

The antimicrobial susceptibility of *E. coli* 47EC was evaluated by determining the MIC values of different kinds of antibiotics, following the Clinical & Laboratory Standards Institute (CLSI, United States) M100 protocol [17]. A total of nine drugs (all purchased from Solabio), including aminoglycosides (gentamicin), carbapenems (meropenem), cephalosporins (ceftazidime), phenicols (chloramphenicol), tetracyclines (tigecycline), fluoroquinolones (ciprofloxacin), polymyxins (polymycin), rifaximin and mitomycin C, were included in the tests. The results were interpreted using the CLSI breakpoint (CLSI M100, 28th Edition). If the CLSI breakpoint was not available, the European Committee on Antimicrobial Susceptibility Testing (EUCAST) breakpoint (v8.1) was used for interpretation. Each antibiotic was tested with three duplicates. *E. coli* ATCC25922 was used for quality control.

### 5.3. Isolation of OMV

OMVs derived from *E. coli* 47EC were isolated and purified according to the previously described method [25]. Mitomycin C has been proven to confer a strong stimulation effect on OMV production, so it was used as the positive control in this study [34,35]. Briefly, *E. coli* 47EC were cultured in 250 mL Luria-Bertani (LB) broth to the middle exponential phase (optical density of 0.5 at 600 nm) at 37 °C and at a speed of 180 rpm. Then, antibiotics were added to the culture at final concentrations of 1/4 MICs and 1/2 MICs. Both the *E. coli* 47EC with and without antibiotics treatment were incubated for 12 h and then used for the isolation of the OMVs. The bacterial cells in the cultures were removed by centrifugation at 4 °C and 10,000 rpm (12,000× *g*) for 10 min, and the supernatants were filtered through a 0.2 µm vacuum filter to remove residual cells and cellular debris. The filtrates were precipitated by ultracentrifugation at 4 °C and 35,000 rpm (100,000× *g*) for 2 h using a 45 Ti rotor (Beckman, Brea, CA, USA). The pellets were washed with phosphate-buffered saline (PBS) (pH 7.4) and ultracentrifugated again to collect the OMVs. The pellet containing the OMVs was resuspended in 1 mL of PBS and sterilized through 0.22 µm filters. The OMVs were verified by culturing aliquots on blood agar to ensure no contamination of bacteria and stored at −80 °C until use.

### 5.4. Quantification of OMVs

The OMV samples (10 µg) were treated with 1 U DNase (Beyotime Biotechnology, Shanghai, China) to remove extravesicular DNA and 100 µg/mL proteinase K (Takara, Shiga, Japan) to digest the phage coats. DNase- and proteinase K-treated OMVs were subsequently quantified using a bicinchoninic acid (BCA) protein assay kit (Beyotime Biotechnology, Shanghai, China), according to the manufacturer’s protocol. 

### 5.5. Size Distribution and Zeta Potential of OMVs

The OMV samples were diluted to a concentration of 20 µg/mL with sterile PBS, and 1 mL of the OMV aliquots was pipetted into a sterile cuvette. The particle size distributions and zeta potentials of the OMVs were detected using Dynamic Light Scattering (DLS) on a Zetasizer Nano ZSE (Malvern Instruments, Worcestershire, UK). All measurements were performed in triplicate at room tempreture.

### 5.6. Detection of tet(X4) in OMVs

The detection of the tigecycline resistant gene of *tet*(X4) in the OMVs was conducted using PCR according to the our previous publication [6]. Real-time quantitative PCR (qPCR) was used to quantify the copy numbers of the *tet*(X4) gene within the OMVs from the antimicrobial-treated groups and the control group. qPCR was conducted using Taq Pro Universal SYBR qPCR Master Mix (Vazyme, Nanjing, China) according to the manufacturer’s instructions. The OMVs were treated with DNase I- and proteinase K first, and then the OMV-DNA was extracted using a Bacterial DNA Extraction Kit (Tiangen Biotech, Beijing, China). The qPCR reaction system includes components of OMVs-DNA (1 µL), 2 × Taq Pro Universal SYBR qPCR Master Mix (10 µL), forward primer (10 µM, 0.4 µL), reverse primer (10 µM, 0.4 µL) and ddH2O (8.2 µL). The primers used for qPCR were *tet*(X4)-qPCR-F: (5′-TTCAATGCTTGCCCACAAGG-3′) and *tet*(X4)-qPCR-R: (5′-ATGAGCAGCATCGCCAATC-3′). The amplification was initially activated at 95 °C for 30 s and then subjected to 40 PCR cycles of denaturation at 95 °C for 15 s and extension and annealing at 60 °C for 1 min, then was heated to 95 °C for 15 s. The standard curves were constructed using constructed recombinant plasmid pUC18-*tet*(X4). The plasmid copy number (*tet*(X4) concentration) within the OMVs was converted in plasmid copy number, using the following equation: DNA (copy) = [6.02 × 10^23^(copy/mol) × DNA amount(g)]/[DNA length(dp) × 660 (g/mol/dp)] [36]. 

### 5.7. Morphology of OMVs

Among all the tested antibiotics, ciprofloxacin exhibited the strongest stimulation effect on the production of OMVs. Thus, the OMVs from the ciprofloxacin-treated group and the antibiotic-free group were compared for their morphology by using a transmission electron microscope (TEM). The OMV sample was dropped on a copper grid, stained with 1% phosphotungstic acid and then observed using a Tecnai high-field transmission electron microscope (Phillips company, Amsterdam, Holland).

### 5.8. Transcriptome Analysis of E. coli 47EC under Ciprofloxacin Treatment

The ciprofloxacin-treated *E. coli* 47EC were subjected to transcriptome sequencing to analyze the underlying mechanism of the antibiotic-induced formation of the OMVs. The 47EC cultures at OD_600_ 0.5 were incubated with a 1/2 MIC concentration of ciprofloxacin for 6 h. Then, the total RNA was isolated using the Trizol Reagent (Invitrogen Life Technologies, Carlsbad, CA, USA), and the Zymo-Seq RiboFree Total RNA Library Kit was used to remove the rRNA from the total RNA. Sequencing libraries were generated using the AMPure XP system (Beckman Coulter, Beverly, CA, USA) and sequenced on a NovaSeq 6000 platform (Illumina, San Diego, CA, USA) by the Shanghai Personal Biotechnology Cp. Ltd. The raw data were filtered using fastp (0.22.0) software and the clean data were mapped to the reference genome using Bowtie2. v2.5.1. HTSeq (v0.9.1) was used to quantify the original expression level of the gene. FPKM (fragments per kilobaseof exon per million fragments mapped) was used to normalize the expression. DEGSeq (v1.38.3) was conducted to analyze the differential expressed mRNA; transcripts with |log2FoldChang| > 1 and *p*-value < 0.05 were considered as differentially expressed mRNA. Bidirectional cluster analysis on all mRNA and samples was performed using the R language Pheatmap (v1.0.12) software package. The Euclidean method was used to calculate the distance, and the hierarchical clustering longest-distance method was used to accomplish the clustering. GO enrichment analysis on differential genes was performed using topGO; *p*-value was calculated by the hypergeometric distribution method (the standard of significant enrichment is *p*-value < 0.05). ClusterProfiler (v4.6.0) software was used to carry out the enrichment analysis of the KEGG pathway of the differential genes, focusing on the significant enrichment pathway with *p*-value < 0.05. The ggplot2 package was used for figure drawing. Raw and processed data of the RNA-seq have been submitted to the Sequence Read Archive (SRA) at the National Center for Biotechnology Information (NCBI) under accession no. PRJNA1049989.

qRT-PCR was performed on five genes to validate the transcriptome data. The qRT-PCR was performed using the RNA extracted under similar conditions to those of theRNA-seq analysis. cDNA was synthesized using the HiScript II 1st Strand cDNA Synthesis Kit (+gDNA wiper) (Vazyme, Nanjing, China) according to the manufacture’s instructions. Primers were designed using Primer3Plus (https://www.primer3plus.com/index.html, accessed on 12 December 2023) (Table 4) and the 16S rRNA gene was used as the housekeeping control. All qRT-PCR reactions were run on the LightCycler 480 real-time PCR system (Roche, Indianapolis, IN, USA) using the Taq Pro Universal SYBR qPCR Master Mix (Vazyme, Nanjing, China) according to the manufacture’s protocol. qRT-PCR was performed with three biological replicates for each sample. The relative expression of each gene was determined by the 2^−ΔΔct^ method.

## Figures and Tables

**Table 1:** MICs of the drugs against *E. coli* 47EC and ATCC25922.

Antimicrobial	MICs (μg/mL) for	
	*E. coli* 47EC	ATCC25922
Rifaximin	256.000	16.000
Tigecycline	16.000	0.125
Ceftazidime	0.250	0.250
Ciprofloxacin	1.000	0.008
Meropenem	0.030	0.031
Chloramphenicol	256.000	4.000
Polymyxin B	0.125	0.500
Gentamicin	8.000	1.000
Mitomycin C	16.000	1.000

**Table 2:** The copy numbers of *tet*(X4) in the OMVs from *E. coli* 47EC under exposure to antimicrobials at different concentrations.

Antimicrobial	Copies of *tet*(X4) Gene in OMVs from *E. coli* 47EC at Different Conditions (Copies/µL)		
	Without Antimicrobial	1/4 MIC	1/2 MIC
Control	2.937 × 10^4^		
Mitomycin C		1.757 × 10^5^	6.875 × 10^5^
Ciprofloxacin		1.657 × 10^5^	5.769 × 10^5^
Meropenem		2.488 × 10^5^	7.783 × 10^4^
Tigecycline		4.125 × 10^4^	1.500 × 10^4^
Ceftazidime		3.226 × 10^5^	5.890 × 10^5^
Gentamicin		5.471 × 10^5^	2.478 × 10^5^
Rifaximin		1.755 × 10^3^	2.867 × 10^4^
Chloramphenicol		3.067 × 10^5^	1.384 × 10^5^
Polymycin		3.954 × 10^5^	8.425 × 10^4^

**Table 3:** Differentially regulated genes in ciprofloxacin-treated *E. coli* 47EC associated with SOS response and prophage.

Gene No.	Gene Name	Product Name	Gene Start	Gene End	RNA-Seq Fold Change	Adjusted *p* Value
gene-PQQ28_RS02015	*rtcB*	RNA-splicing ligase RtcB	351,645	352,871	6.616	1.43 × 10^−3^
gene-PQQ28_RS00805	*dinD*	DNA damage-inducible protein D	86,763	87,587	15.868	2.47 × 10^−13^
gene-PQQ28_RS06340	*recA*	DNA recombination and repair protein RecA	1,229,187	1,230,248	28.095	5.40 × 10^−14^
gene-PQQ28_RS06345	*recX*	Recombination regulator RecX	1,230,317	1,230,817	27.885	3.46 × 10^−14^
gene-PQQ28_RS06590	*recN*	DNA repair protein RecN	1,270,949	1,272,610	51.591	3.16 × 10^−5^
gene-PQQ28_RS10705	*ruvA*	Holliday junction ATP-dependent DNA helicase RuvA	2,197,626	2,198,237	2.2830	1.90 × 10^−2^
gene-PQQ28_RS10770	*yebG*	DNA damage-inducible protein YebG	2,212,855	2,213,145	10.505	5.99 × 10^−5^
gene-PQQ28_RS11320	*cho*	Excinuclease Cho	2,317,027	2,317,914	4.024	4.23 × 10^−2^
gene-PQQ28_RS11380	*ydjM*	Inner membrane protein YdjM	2,329,376	2,329,966	4.493	4.49 × 10^−2^
gene-PQQ28_RS14070	*umuC*	DNA polymerase V catalytic protein	2,872,722	2,873,990	6.838	7.53 × 10^−7^
gene-PQQ28_RS14075	*umuD*	Translesion error-prone DNA polymerase V autoproteolytic subunit	2,873,990	2,874,409	12.589	8.69 × 10^−11^
gene-PQQ28_RS15315	*dinI*	DNA damage-inducible protein I	3,067,149	3,067,394	8.974	4.42 × 10^−5^
gene-PQQ28_RS16320	*sulA*	Cell division inhibitor SulA	3,263,018	3,263,527	32.872	8.06 × 10^−7^
gene-PQQ28_RS17075	*dinG*	ATP-dependent DNA helicase DinG	3,436,699	3,438,849	3.112	1.16 × 10^−3^
gene-PQQ28_RS17175	*uvrB*	Excinuclease ABC subunit B	3,455,919	3,457,940	5.136	4.98 × 10^−6^
gene-PQQ28_RS19460	*yafP*	GNAT family N-acetyltransferase	3,933,638	3,934,090	8.469	5.61 × 10^−3^
gene-PQQ28_RS19465	*dinB*	DNA polymerase IV	3,934,087	3,935,142	5.458	4.21 × 10^−6^
gene-PQQ28_RS20795	*polB*	DNA polymerase II	4,218,200	4,220,551	7.983	1.56 × 10^−8^
gene-PQQ28_RS23100	*ssb1*	Single-stranded DNA-binding protein	4,690,573	4,691,109	3.367	1.96 × 10−2
gene-PQQ28_RS23105	*uvrA*	Excinuclease ABC subunit UvrA	4,691,364	4,694,186	5.920	6.92 × 10^−7^
gene-PQQ28_RS23255	*lexA*	Transcriptional repressor LexA	4,728,041	4,728,649	8.816	2.20 × 10^−9^
gene-PQQ28_RS24565	*uvrD*	DNA helicase II	5,014,795	5,016,957	2.963	6.24 × 10^−3^
gene-PQQ28_RS01710	*dcrB*	Phage sensitivity protein DcrB	287,181	287,738	2.643	4.40 × 10^−3^
gene-PQQ28_RS14425	PQQ28_RS14425	Phage NinB-Orf DNA recombination	2,924,894	2,925,334	586.660	4.67 × 10^−2^
gene-PQQ28_RS14905	PQQ28_RS14905	Phage protein	2,993,478	2,993,741	352.651	4.72 × 10^−2^

**Table 4:** RT-qPCR primers used in this study.

Target Genes	Primers	Primer Sequences (5′-3′)	Length (bp)
*recA*	*recA*-F	GGCGTCGATATCGACAACCTG	138
	*recA*-R	CGCTTTCGGCGTCAGTGC	
*cho*	*cho*-F	GGATGAAGCCGCCATGCTAC	94
	*cho*-R	GATTAATCGCGCTTCAAGG	
*lexA*	*lexA*-F	TCATCCGTGATCACATCAGC	113
	*lexA*-R	TGCCAGCGCCTTCAGATG	
*dcrB*	*dcrB*-F	CATGCATGTCTGGTCCGACG	133
	*dcrB*-R	TTGCAGCTGCGGATCGCG	
PQQ28-RS14425	PQQ28-RS14425-F	CACTACCCATCGACGACAAG	98
	PQQ28-RS14425-R	ATCGTTCAGCATCGGCCAC	

## Data Availability

The original contributions presented in the study are included in the article, further inquiries can be directed to the corresponding author/s.
